# A Multi-Institutional Collaboration to Understand Neoplasia, Treatment and Survival of Snakes

**DOI:** 10.3390/ani12030258

**Published:** 2022-01-21

**Authors:** Elizabeth G. Duke, Scott H. Harrison, Anneke Moresco, Tim Trout, Brigid V. Troan, Michael M. Garner, Madison Smith, Sidney Smith, Tara M. Harrison

**Affiliations:** 1 Department of Clinical Sciences, North Carolina State University College of Veterinary Medicine, Raleigh, NC 27607, USA; ecgraebe@ncsu.edu (E.G.D.); brigid@troan.org (B.V.T.); 2 Exotic Species Cancer Research Alliance, North Carolina State University College of Veterinary Medicine, Raleigh, NC 27607, USA; moresco2@gmail.com (A.M.); mrsmit18@ncsu.edu (M.S.); slsmit16@ncsu.edu (S.S.); 3 Department of Biology, North Carolina Agricultural and Technical State University, Greensboro, NC 27514, USA; scotth@ncat.edu; 4 Department of Animal Care, Denver Zoo, Denver, CO 80205, USA; timtrout@comcast.net; 5 Northwest ZooPath, Monroe, WA 98272, USA; zoopath1@gmail.com

**Keywords:** Boidae, chemotherapy, Colubridae, neoplasia, oncology, surgery, prevalence, Viperida

## Abstract

**Simple Summary:**

Multiple studies have focused on types of neoplasia found in snakes, but an overall estimation of prevalence including total populations of animals at multiple facilities has not been conducted. Additionally, an in-depth evaluation of methods of therapy and survival of snakes with neoplasia has not been carried out. This study calculated the prevalence of tumors in 133 snakes, representing 65 different species, housed in six zoos and aquariums. Survival times were evaluated to determine whether these snakes were more likely to die from their tumors versus another cause. Treatment outcomes were evaluated to determine if the used treatment types lengthened the snakes’ life spans. Common or northern watersnakes (*Nerodia sipedon*), eastern diamond-backed rattlesnakes (*Crotalus adamanteus*), and timber rattlesnakes (*Crotalus horridus*) had the highest prevalence of tumors. Malignant (cancerous) tumors predominated, and the snakes with these tumors were significantly more likely to die of their cancer than those with benign tumors. Thirty-six of the 133 snakes received treatment for their tumors. There was no significant difference in survival times for those treated and not treated. This population is a subset of the overall snake population under managed human care, and a larger collection of snake tumor and population data could yield different results. Therefore, additional snake cases, along with other non-domestic species, are continuing to be curated in a database (Exotic Species Cancer Research Alliance tumor database). The goal of this data collection is to provide data on a select population of snakes to help veterinarians gain greater understanding of cancer types and to treat cancer in these animals.

**Abstract:**

This multi-institutional collaborative study of neoplasia in snakes reviewed medical records of snakes at each facility to determine species prevalence, survival, and methods of treatment. Complete species numbers of snakes were also collected at each facility. In total, 65 species, 133 snakes, and 149 unique neoplasias were included in this study. Affected species, age, sex, and their tumor prevalence, tumor type and location, metastasis, treatment, and survival data are reported. The highest species-specific tumor prevalence was in Common or Northern Watersnakes (*Nerodia sipedon*) (30.8%, *n* = 4 of 13), Eastern Diamond-Backed Rattlesnakes (*Crotalus adamanteus*) (26.3%, *n* = 5 of 19), and Timber rattlesnakes (*Crotalus horridus*) (22.7%, *n* = 5 of 22). Malignant tumors predominated (86.6%, *n* = 129 of 149) with soft tissue sarcomas being the most common (30.2%, *n* = 45 of 149). Snakes with malignant neoplasia, metastases, or indeterminate presence of metastases were statistically more likely to die from their neoplasms than snakes having either benign neoplasia or no diagnosed metastases (*p* < 0.05). Gender, taxonomic family, and species of those evaluated did not significantly affect the outcome of snakes with neoplasia. Only 27.1% (*n* = 36 of 133) of snakes received a reported form of treatment and, for those treated, surgical excision was the most common treatment modality. There was not a significant difference in outcome based on treatment; however, surgery and chemotherapy were associated with death from a cause other than their tumor.

## 1. Introduction

Research involving neoplasia in snakes is not new and in fact has occurred over numerous years involving evaluations of medical cases at the London Zoo, the Registry of Tumors of Lower Animals [1,2,3], and individual laboratories or facilities [4,5,6]. Overall, these previous studies have found that neoplasia appears to be common in snakes [7,8], and it is well shown in the literature that snakes tend to have higher prevalence of cancer reported than other reptiles [7,9]. However, these publications on snake neoplasia have typically focused only on the types of neoplasia diagnosed [10,11], the prevalence of neoplasia at a single institution or laboratory [4,12], or on an individual animal’s treatment [13,14]. A type of multi-institutional study on museum specimens has been done as well as a literature review, however, this study was never able to confirm the presence of neoplasia due to the lack of confirmatory testing [15]. Other studies do exist on reporting snake neoplasia prevalence within a group of snakes, including cutaneous chromatophoromas [11,16,17,18,19]. One of these previous publications evaluated the prevalence of neoplasia from cases submitted to a diagnostic laboratory and they found that the prevalence was highest in Crotalids, Viperids, and Boids [7]. This study, however, only looked at the samples submitted to their laboratory and was unable to evaluate the prevalence based on the populations of animals from where they originated. Additionally, previous studies found that malignant tumors are most commonly reported in snakes, and that they were mesenchymal or epidermal in origin [4,6,7,20].

Previous published literature also is lacking on evaluation of treatments and survival times. Although papers exist on treatments in individual snakes, as well as a report on treatment of other snakes, there is no mention of overall survival time [13,21,22]. Currently, even though snake metabolism is very different from mammal metabolism, current predictions of a snake’s survival with neoplasia are based on survival time in domestic dogs and cats with cancer due to a lack of survival studies in snakes.

The present study therefore focused on (a) investigating the prevalence of neoplasia for snakes housed at six institutions using the combined total population for each snake species at these institutions, and (b) determining if a snake with neoplasia was more likely to die or be euthanized due to neoplasia versus another cause in order to evaluate survival for snakes with neoplasia.

## 2. Materials and Methods

### 2.1. Case Selection

Six institutions participated in this study. Complete medical records were obtained for snakes that had a biopsy or necropsy report with a histologic diagnosis of neoplasia. Cases were confirmed to be neoplasia by board-certified veterinary pathologists working at either independent laboratories or university settings. Cases were confirmed to be neoplasia based on the diagnosis in the medical record and classified as to current nomenclature by board-certified veterinary pathologists specializing in zoological medicine cases (Troan, Garner). To determine prevalence for each species, the total number of individuals of that species housed at each institution for a given time were gathered from their respective medical record managers or by obtaining species totals from the Species360 Zoological Information Management System (ZIMS). Population totals included all ages and several snakes that were lost to follow-up or that were transferred to other institutions.

### 2.2. Medical Records Review

Medical records of the six participating institutions were reviewed. Animals were categorized taxonomically (family and species), by sex (male, female, unknown), and by maturity (adult; juvenile being <3 months of age; or unknown). Diagnoses were grouped by primary histologic diagnosis and behavior (benign versus malignant). All lymphomas or leukemias were designated as malignant due to the multicentric nature of these neoplasms. Animals that had detectable metastases were categorized in the metastasis category. Treatments were separated into general groups including (1) surgery only, (2) chemotherapy (which included steroid therapies and non-steroidal anti-inflammatory drugs (NSAIDs) used longer than 5 days for postoperative analgesia), (3) surgery and chemotherapy (including steroid and nonsteroidal therapies (as above)), (4) supportive care (which included systemic or topical antibiotics and NSAIDs (as above)), (5) unknown treatment, and (6) no treatment provided. Medical records were collected and managed using REDCap electronic data capture tools hosted at North Carolina State University College of Veterinary Medicine [23,24]. REDCap (Research Electronic Data Capture, Vanderbilt University, Nashville, TN, USA) is a secure, web-based software platform designed to support data capture for research studies, providing (1) an intuitive interface for validated data capture; (2) audit trails for tracking data manipulation and export procedures; (3) automated export procedures for seamless data downloads to common statistical packages; and (4) procedures for data integration and interoperability with external sources.

### 2.3. Data Analysis

Database information was analyzed using IBM Corp. Released 2019. IBM SPSS Statistics for Windows, Version 26.0. Armonk, NY, USA [25] and R statistical computing software (version 3.6.1; R Core Team 2020. R: A language and environment for statistical computing. R foundation for Statistical Computing, Vienna, Austria, https://www.R-project.org/ accessed 18 January 2022) [26]. Prevalence of neoplasia per species was done for those that have greater than 10 total individuals (those with neoplasia and without). Frequencies, survival curve analysis, and boosting analyses were used to evaluate the data. For survival curve analysis, Kaplan–Meier survival curves and log-rank tests were generated with the survminer package (R package version 0.4.1, https://CRAN.R-project.org/package=survminer accessed on 18 January 2022) [27] and survival package (R package version 2.38, https://CRAN.R-project.org/package=survival accessed 18 January 2022) [28]. For the statistical method of boosting, the mboost package (Model-based boosting, R package version 2.6.0, https://CRAN.R-project.org/package=mboost accessed 18 January 2022) [29] was used. Neoplasms were grouped into more general classification categories by behavior (benign vs. malignant) as well as by tissue and tumor type for mboost evaluation of survival of animals affected with these types of neoplasms. For boosting analysis, predictor variables were modeled with the use of btree as a base-learner for decision stumps. The set of eight predictor variables were presence or lack of detectable of metastasis, primary histological diagnosis, type of tumor, number of observed tumor types (1 or 2), type of treatment, taxonomic family, species, and sex. Outcomes were assigned to snakes, with “+1” designated for those that died or were euthanized due to the neoplasm and “0” designated for those that were euthanized due to another cause. Snakes without a known cause of death, or who were lost to follow-up, were excluded from outcome analysis. The modeled effects of the eight predictor variables with outcomes for each animal evaluated were compared to a set of 2000 null model distributions of effects generated from modeling done with permutated outcomes, with *p* < 0.05 being the threshold of significance regarding the tails of the null model distribution (two-sided hypothesis), similar in method to Mayr et al. [30]. Prevalence was calculated only for species with 10 or more individuals in the study population.

## 3. Results

### 3.1. Study Population

A total of 133 snake neoplasia cases representing 65 different species were included. Time spans varied by institution due to medical record accessibility. Time spans were Institution 1: (1985–2018); Institution 2: (2013–2017); Institution 3: (2003–2018); Institution 4: (1974–2018); Institution 5: (1980–2018); and Institution 6: (2002–2018). Participating institutions are located in Colorado, Ohio, North Carolina, and Texas (alphabetical order, see acknowledgements).

### 3.2. Neoplasia Information

There were 149 neoplasms included in the study. Malignant tumors predominated (86.6%, *n* = 129 of 149). Only 13 benign tumors were identified (8.7%, *n* = 13 of 149), and there were 7 unspecified tumors (4.7%, *n* = 7 of 149) as their behavior was not definitive based on histology. The sex distribution consisted of 67 females, 63 males, and 3 of undetermined sex. Of those with benign neoplasia 4 were females and 5 were males. There were 61 females and 56 males with malignant neoplasia and 2 females and 2 males with unspecified benign or malignant neoplasia. The median age at the time of diagnosis was 138 months, and ages ranged from 42–420 months. The average age of animals with benign neoplasms was 176 months (median 141 months) and the average age of those with malignant neoplasms was 150 months (132 median). Seven individuals were of unknown age. Detectable metastasis was reported in 42.9% (*n* = 57 of 133) of snakes with neoplasia. Multi-organ involvement existed in 27 snakes, while 17 snakes had multicentric lymphoma or leukemia (Table 1).

### 3.3. Neoplasia Prevalence

The neoplasia prevalence by taxonomic family (including only those species with 10 or more individuals) ranged from 3.3–7.3% and was not significantly different (χ^2^ = 1.88, *p* = 0.17) among the various families.

The highest prevalence of neoplasia by species, was in the common or northern watersnake (*Nerodia sipedon*) (30.8%, *n* = 4 of 13) followed by the eastern diamond-backed rattlesnake (*Crotalus adamanteus*) (26.3%, *n* = 5 of 19) and the timber rattlesnake (*Crotalus horridus*) (22.7%, *n* = 5 of 22) (Table 1). The species with the lowest prevalence was the plains gartersnake (*Thamnophis radix*) (0.8%, *n* = 4 of 500). Both, the species with the highest neoplasia prevalence (the watersnake) and the species with the lowest prevalence (the plains gartersnake) were from the *Colubridae* family of snakes. The prevalence of neoplasia was significantly different between these two species, (χ^2^ = 74.13, *p* < 0.01). Prevalence of neoplasia and total number of snakes per institution are listed in Table 2.

### 3.4. Treatment

Treatment for neoplasia was administered to 27.1% (*n* = 36 of 133) of the snakes. Of the 36 snakes that were treated, a survival time was reported in all but three snakes which were euthanized during surgery (Table 3 includes the 33 snakes that reported a survival time). The median survival time was 5.5 months (range 0.25–108 months). The main treatment modality used was surgery (complete or incomplete excision) (97.2%, 35 of 36). Surgery in combination with chemotherapy was employed in three snakes, and surgery and radiation were performed in two snakes. Surgery, chemotherapy, and radiation were all used to treat one snake, and chemotherapy alone was used to treat one snake. Supportive care in the form of antibiotics, fluids, or NSAIDs was used in eight snakes in addition to a treatment modality and used in four snakes without other treatment modalities (Table 3).

### 3.5. Outcome and Survival

Outcome effects and significance of predictors were estimated and evaluated through boosting and permutation as described, with the outcome effects representing estimates of coefficients in a generalized linear model for death due to tumor, or death due to another cause. Animals with malignant neoplasms were significantly more likely to die of their cancer (*p* < 0.05) and snakes with benign neoplasms were significantly less likely to die of their cancer (a cause of death other than tumor) (*p* < 0.5). Chromatophoromas were identified as a neoplasm significantly associated with a cause of death other than tumor (*p* < 0.05). Snakes without metastases were also significantly associated with a non-tumor-related cause of death (*p* < 0.05). Snakes with metastases or an unknown condition of metastasis were significantly associated with a tumor-related cause of death (*p* < 0.05). Number of unique tumors, species and sex were not significantly associated with a cause of death due to neoplastic disease and had absolute values of outcome effect <0.01, with the exception of the *Thamnophis radix* species having an outcome effect of 0.014. Significant outcome effects for predictor variable values are shown in Table 4.

**Figure 1:**
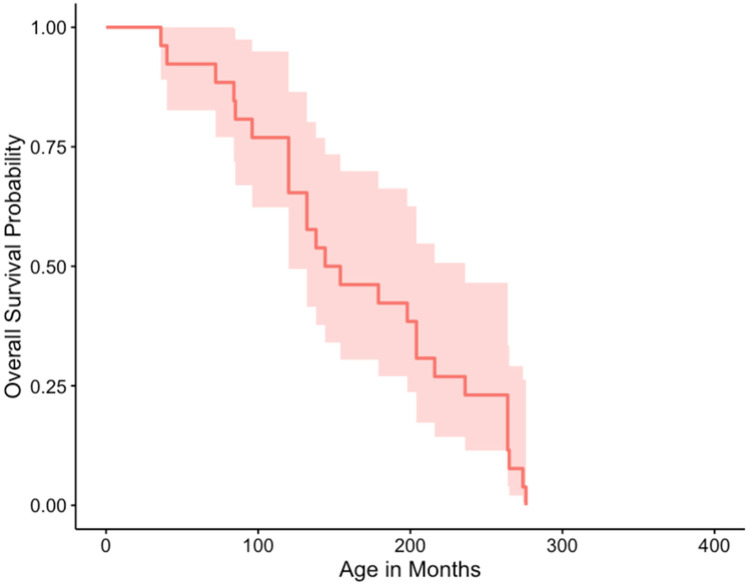
Overall survival curve of snakes treated for their neoplasia.

**Figure 2:**
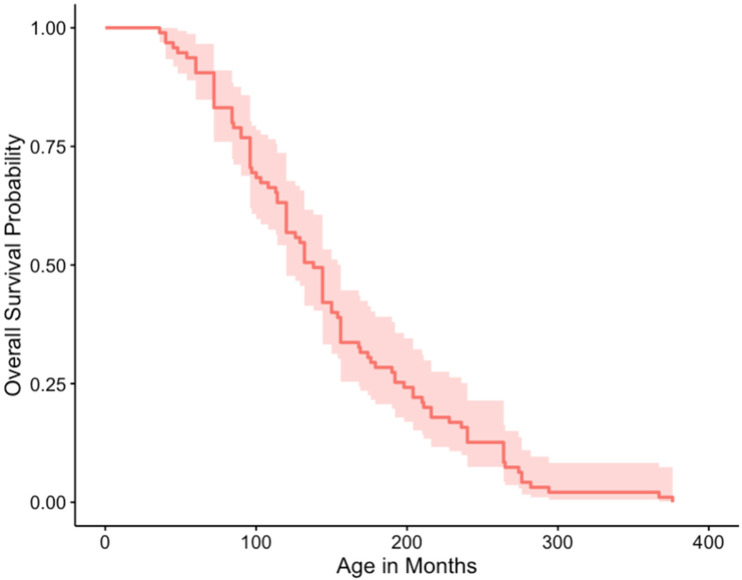
Overall survival curve of snakes not treated for their neoplasia.

Cases for other modalities other than surgery were too low to analyze separately, therefore cases treated with multiple modalities (surgery, chemotherapy, and/or radiation) were grouped. Median survival times for no treatment, surgery only, and multiple modalities were 1, 5.5, and 13 months respectively. Survival curves plotted for those snakes that were treated and those that were not, were not significantly different based on a log-rank test (*p* = 0.3) (Figure 1 and Figure 2).

## 4. Discussion

By collecting data from multiple institutions, this study reaffirmed that snakes have a tendency to develop malignant neoplasia indicating that histological sampling of masses may be key to early diagnosis and more successful treatment. Additionally, this study calculated species specific prevalence for 65 species and specifically for 44 species in which more than 10 individuals were included which were used to calculate overall family prevalence. The reason to only calculate prevalence for 10 or more individuals was to reduce the potential for inaccurate levels of prevalence to be reported for those species having low numbers of animals. Although this study evaluated neoplasia in 65 species, we were not able to evaluate the prevalence of neoplasia in all snakes due to which species were present at the six institutions evaluated. Therefore, results of this study can only be applied to those that were evaluated and included in this study. It is possible that a potential variation in necropsy techniques or time of death prior to sampling could have impacted the ability to diagnose neoplasia at certain facilities, but we attempted to minimize this through involving multiple facilities. Finally, snakes that received no treatment had lower survival times than those that were treated. This may have been because some snakes showed advanced disease at the time of first diagnosis and therefore were not considered suitable candidates for treatment, or those snakes may have been diagnosed at the time of death due to their cancer and as such would make their survival times lower.

By including both neoplasia cases rising to the level of treatment along with the non-affected population, the inclusion of the latter category by this study has helped address publication bias that has been oriented toward the former. This, further addresses laboratory submission bias as published reports and submitted cases preferentially include cases that are interesting, unique, or survive due to a certain therapy. We also avoided regional bias by including cases from multiple institutions across the United States. This collection of cases from multiple areas also helps to minimize a genetic component. More charismatic, larger or more colorful snakes can sometimes be chosen to be exhibited, which can skew the number of individuals representing a given species of snakes. This bias was likely reduced by using multiple institutions in different areas with different-sized facilities that, together, are less likely to ascribe to any singular modality for choosing a snake species.

The present study documented that the most prevalent neoplasms in snakes were malignant mesenchymal tumors of the skin and soft tissue (*n* = 45 of 149), malignant tumors of the alimentary system/liver/gallbladder (*n* = 31 of 149), malignant tumors of mast cell/hemolymphatic/histocytic origin (*n* = 18 of 149), and malignant tumors of the urinary/genital systems (*n* = 15 of 149).

We did not find any statistically significant difference in prevalence between families. There was a statistically significant difference in prevalence by species.

We did not find any differences in prognostic outcome between families or genera. These families and genera typically range in size and length. There could potentially be a bias introduced that with smaller or skinnier snakes it may be easier to observe neoplasia than with larger or wider diameter snakes, but as the length and width of the snakes was not routinely recorded in the medical records, it was not able to be evaluated.

It is not surprising that those animals with malignant, metastatic, or indeterminant metastatic neoplasia were more likely to die from the tumor than those with benign or non-metastatic neoplasms. Malignant and metastatic neoplasms tend to negatively affect multiple organ systems, which explains the negative outcome. The only neoplasm that did not contribute significantly to the death of the animal in our data was a chromatophoroma, which is different from previously published literature [16].

Treatment was not commonly performed in the snakes that we evaluated, possibly because very early clinical signs of neoplasia may be limited to subtle changes in behavior or physical appearance which may be difficult to detect during routine husbandry observations. These behavioral cues can include, but are not limited to, changes in feeding behavior, odd body positioning, and increases or decreases in activity [31,32]. External physical changes often are dermal abnormalities such as frequent and/or incomplete ecdysis, areas of discoloration or infection, cutaneous or intracoelomic swellings, or weight changes inconsistent with body condition [21]. Finally, clinical signs may not be observable until widespread (metastatic) or advanced disease. With continued, improved frequency of physical examinations in zoological facilities as well as in those kept as pets and with continued use of diagnostic imaging such as ultrasound, radiographs, and computed tomography (CT) scans, this could aid in diagnosing these neoplasias at an earlier stage prior to metastasis and may allow for additional diagnostics such as fine needle aspirates, biopsies, or surgical removal of masses.

Consistent with previous reports, surgical excision was the most common treatment modality and showed an increase in survival time in certain cases [13,33]. Additionally, a previous study found that even removing a portion of a neoplastic mass had a positive effect on survival, which appeared to be similar to results in this study [34]. Specifically, a cornsnake with a surgically removed soft tissue sarcoma without metastasis had the longest survival time of 108 months compared to other snakes that received some form of treatment specifically for their neoplasia. In general, the only other therapies that had a survival time of greater than 22 months were surgical or surgery and chemotherapy treatments (survival time 84 months). This survival time exceeds that of two other snakes treated with chemotherapy and surgery (5.5 and 20 months) as well as snakes treated with chemotherapy only (11 months) or surgery, chemotherapy, and radiation (12 months). Prior reports of published case reports on radiation and chemotherapy treatments in snakes, documents an increase in survival time for some patients when treated with radiation and chemotherapy as sole treatment therapies, or when used in combination with surgery [3,35]. It must be noted, however, that longer survival times may not necessarily correlate with improved quality of life due to potential side effects of chemotherapy and radiation. Many medical or radiation treatment protocols were extrapolated from domestic animal dosages and frequency schedules, which may not be appropriate for snakes and could account for the lower survival times. Overall, no treatment method demonstrated a statistically significant improvement in prognosis, but this may be due to the low number of treated animals in this study or to the treatment protocols not being tailored to snakes or their neoplasia type. As more frequent examinations occur and diagnostic techniques improve, more cases will be diagnosed and potentially treated, thereby improving our knowledge about neoplastic disease, treatment, and survival in these species.

## 5. Conclusions

Our data show that snakes have a moderate risk of developing neoplasia, but when they do, it is likely malignant. Surgical excision patients survive longer than patients that received no treatment. Modalities such as chemotherapy and radiation need further refinement to result in prolonged survival times compared to surgical removal only. However, relatively few cases of snakes with cancer were treated in this study. Although data and results collected for this study represent multiple institutions and reduce bias, furthering knowledge of neoplasia prevalence, behavior, and treatment in snakes will benefit from a larger aggregated dataset (www.escra.org (accessed on 18 January 2022)), in particular a larger number of treated cases.

## Figures and Tables

**Table 1:** Tumor Type and Prevalence by Species.

Family	Species and Prevalence	Common Name	Benign	Malignant	Unspecified
Boidae	*Acrantophis dumerili* 2.4% (1/41)	Dumeril’s Ground Boa		(1) Soft Tissue Sarcoma	
	*Boa constrictor* 14.3% (2/14)	Boa Constrictor		(1) Neuroendocrine Tumor (1) Soft Tissue Sarcoma	
	*Chilabothrus granti* 7.7% (1/13)	Virgin Islands Boa		(1) Thyroid Carcinoma (1) Hepatocellular Carcinoma	
	*Eunectes murinus* 4.3% (1/23)	Green Anaconda			(1) Sertoli Cell Tumor
	*Lichanura trivirgata* 15.8% (3/19)	Rosy Boa	(1) Pancreatic Adenoma	(1) Undifferentiated Carcinoma (1) Histiocytic Sarcoma	
	*Sanzinia madagascariensis* 20.0% (1/5)	Madagascar Tree Boa		(1) Soft Tissue Sarcoma	
Colubridae	*Coelognathus radiatus* 4% (4/101)	Radiated Ratsnake		(2) Hepatocellular Carcinoma (1) Lymphoma/Leukemia (1) Renal Adenocarcinoma	
	*Elaphe taeniura* 11.1% (1/9)	Taiwan Beauty Snake		(1) Soft Tissue Sarcoma	
	*Heterodon nasicus* 10.3% (3/29)	Western Hog-Nosed Snake		(2) Hepatocellular Carcinoma (1) Soft Tissue Sarcoma	
	*Heterodon platirhinos* 25.0% (2/8)	Eastern Hog-Nosed Snake		(1) Soft Tissue Sarcoma (1) Malignant Chromatophoroma (unspecified)	
	*Lampropeltis getula* 17.9% (5/28)	Kingsnake	(1) Renal Adenoma	(3) Soft Tissue Sarcoma (1) Renal Carcinoma (1) Hepatic Adenocarcinoma	(1) Chromatophoroma (iridophoroma)
	*Lampropeltis triangulum* 1.8% (1/56)	Eastern Milksnake		(1) Gastrointestinal Adencocarcinoma	
	*Leioheterodon madagascariensis* 1.3% (1/78)	Madagascar Giant Hognose Snake		(1) Osteosarcoma	
	*Nerodia sipedon* 30.8% (4/13)	Common Or Northern Watersnake		(3) Soft Tissue Sarcoma (1) Malignant Chromatophoroma (iridophoroma) (1) Malignant Chromatophoroma (Uncharacterized)	
	*Oreocryptophis porphyraceus* 5.3% (1/19)	Black-Banded Trinket Snake		(1) Hepatocellular Carcinoma	
	*Pantherophis guttatus* 9.6% (11/115)	Corn snake		(1) Granulosa Cell Tumor (1) Lymphoma/Leukemia (7) Soft Tissue Sarcoma (1) Osteosarcoma (2) Hemangiosarcoma	
	*Pantherophis obsoletus* (Or) *alleghaniensis* 7.4% (14/189)	Ratsnake		(5) Soft Tissue Sarcoma (1) Gastrointestinal Adencocarcinoma (1) Renal Carcinoma (1) Chondrosarcoma (4) Lymphoma/leukemia (1) Osteosarcoma	(1) Granulosa Cell Tumor
	*Pituophis catenifer* 5.4% (2/37)	Gophersnake (or) Bullsnake		(2) Hepatobiliary Carcinoma	
	*Pituophis lineaticollis* 25.0% (1/4)	Middle American Gophersnake		(1) Undifferentiated Carcinoma	
	*Pituophis ruthveni* 2.4% (1/42)	Louisiana Pinesnake		(1) Soft Tissue Sarcoma	
	*Rhamphiophis oxyrhynchus* 14.3% (1/7)	Rufous-Beaked Snake		(1) Neuroendocrine Tumor	
	*Rhinocheilus lecontei* 16.7% (1/6)	Long-Nosed Snake		(1) Gastrointestinal Adencocarcinoma	
	*Rhynchophis boulengeri* 5.3% (1/19)	Rhinoceros Snake		(1) Lymphoma/Leukemia	
	*Salvadora bairdi* 4.8% (1/21)	Baird’s Patchnose Snake		(1) Renal Adenocarcinoma	
	*Thamnophis cyrtopsis* 100.0% (1/1)	Black-Necked Gartersnake		(1) Malignant Chromatophoroma (Melanoma)	
	*Thamnophis exsul* 10.5% (2/19)	Exiled Gartersnake	(2) Biliary Cystadenoma		
	*Thamnophis radix* (4) 0.8% (4/500)	Plains Gartersnake		(1) Malignant Chromatophoroma (Melanoma) (1) Granulosa Cell Tumor (1) Undifferentiated Carcinoma (1) Hepatocellular Carcinoma	
	*Thamnophis sirtalis* 100.0% (1/1)	Common Gartersnake	(1) Biliary Cystadenoma	(1) Squamous Cell Carcinoma	
	*Trimorphodon lambda* 25.0% (1/4)	Sonoran Lyresnake		(1) Renal Carcinoma	
Elapidae	*Hemachatus haemachatus* 14.3% (2/14)	Ringhals Cobra		(1) Lymphoma/Leukemia (1) Gastrointestinal Adencocarcinoma	
	*Hydrodynastes gigas* 1.4% (1/72)	False Water Cobra		(1) Renal Adenocarcinoma (1) Pancreatic Adenocarcinoma	
	*Naja pallida* 3.8% (1/26)	Red Spitting Cobra		(1) Renal Adenocarcinoma	
	*Ophiophagus hannah* 15.4% (2/13)	King Cobra	(1) Granulosa Cell Tumor	(1) Lymphoma/Leukemia (1) Undifferentiated Adenocarcinoma	
Pythonidae	*Antaresia childreni* 2.3% (1/44)	Children’s Python		(1) Lymphoma/Leukemia (1) Soft Tissue Sarcoma	
	*Liasis mackloti* 50.0% (1/2)	Macklot’s Python		(1) Lymphoma/Leukemia	
	*Malayopython reticulatus* 25.0% (1/4)	Reticulated Python		(1) Hepatocellular Carcinoma	
	*Morelia spilota* 12.5% (1/8)	Carpet Python		(1)Esophageal Carcinoma	
	*Morelia viridis* 1.7% (1/59)	Green Tree Python		(1) Squamous Cell Carcinoma	
	*Python bivittatus* 12.5% (1/8)	Burmese Python		(1) Soft Tissue Sarcoma	
	*Python regius* 10.5% (2/19)	Ball Python		(1) Squamous Cell Carcinoma (1) Gastrointestinal Adenocarcinoma	(1) Chromatophoroma (melanophoroma)
Viperidae	*Agkistrodon contortrix* 37.5% (3/8)	Copperhead	(2) Hepatocellular Adenoma	(1) Lymphoma/Leukemia	
	*Agkistrodon piscivorus* 18.2% (2/11)	Cottonmouth		(1) Soft Tissue Sarcoma (1) Lymphoma/Leukemia	
	*Atheris squamigera* 7.4% (2/27)	African Bush Viper		(1) Hepatic Cystadenocarcinoma (1) Oviduct Adenocarcinoma	
	*Bitis arietans* 50.0% (2/4)	Puff Adder		(1) Renal Carcinoma (1) Soft Tissue Sarcoma	
	*Bitis nasicornis* 5.9% (3/51)	Rhinoceros Viper		(1) Renal Adenocarcinoma (1) Cholangiocellular carcinoma (1) Renal Carcinoma (1) Lymphoma/Leukemia	
	*Bothriechis rowleyi* 4.2% (2/48)	Rowley’s Palm Pit Viper		(1) Soft Tissue Sarcoma (1) Squamous Cell Carcinoma	
	*Bothriechis schlegelii* 4.4% (2/45)	Eyelash Viper		(2) Soft Tissue Sarcoma	
	*Bothrops asper* 2.4% (1/41)	Terciopelo	(1) Renal Cystadenoma	(1) Soft Tissue Sarcoma	
	*Crotalus adamanteus* 26.3% (5/19)	Eastern Diamond-Backed Rattlesnake		(2) Soft Tissue Sarcoma (1) Lymphoma/Leukemia (1) Squamous Cell Carcinoma (1) Hemangiosarcoma	(1) Sertoli Cell Tumor
	*Crotalus atrox* 9.5% (2/21)	Western Diamond-Backed Rattlesnake		(1) Renal Adenocarcinoma (1) Cholangiocellular Carcinoma	(1) Sertoli Cell Tumor
	*Crotalus cerastes* 20.0% (1/5)	Sidewinder	(1) Hepatic Adenoma		(1) Chromatophoroma (Melanophoroma)
	*Crotalus culminatus* 3.6% (2/55)	Northwestern Neotropical Rattlesnake		(1) Soft Tissue Sarcoma (1) Biliary Adenocarcinoma	
	*Crotalus horridus* 22.7% (5/22)	Timber Rattlesnake	(1) Lipoma	(3) Soft Tissue Sarcoma (1) Ovarian Carcinoma	
	*Crotalus lepidus* 14.3% (1/7)	Rock Rattlesnake	(1) Hepatic Adenoma	(1) Malignant Chromatophoroma (Melanophoroma)	
	*Crotalus molossus* 20.0% (1/5)	Black-Tailed Rattlesnake		(1) Soft Tissue Sarcoma (1) Hepatocellular Carcinoma	
	*Crotalus viridis* 20.0% (1/5)	Prairie Rattlesnake		(1) Hepatocellular Carcinoma (1) Soft Tissue Sarcoma	
	*Lachesis muta* 15.4% (2/13)	South American Bushmaster		(2) Hepatocellular Carcinoma	
	*Montivipera raddei* 7.7% (1/13)	Armenian Viper		(1) Pancreatic Carcinoma	
	*Protobothrops flavoviridis* 16.7% (1/6)	Habu		(1) Soft Tissue Sarcoma	
	*Sistrurus catenatus* 25.0% (1/4)	Eastern Massasauga		(1) Osteosarcoma	
	*Sistrurus miliarius* 14.3% (2/14)	Pygmy Rattlesnake	(1) Pancreatic Adenoma	(1) Biliary Adenocarcinoma	
	*Trimeresurus flavomaculatus* 10.0% (1/10)	Philippine Pit Viper		(1) Lymphoma/Leukemia	
	*Trimeresurus mcgregori* 2.3% (1/44)	McGregor’s Tree Viper		(1) Soft Tissue Sarcoma	
	*Trimeresurus sumatranus* 1.4% (1/74)	Sumatran Pit Viper		(1) Lymphoma/Leukemia	
	*Vipera transcaucasiana* 5.3% (1/19)	Transcaucasian Long-Nosed Viper		(1) Soft Tissue Sarcoma	

**Table 2:** Prevalence of neoplasia at each participating institution.

Institution	Total Snakes with Neoplasia	Total Number of Snakes of Affected Species	Number of Different Affected Species	Prevalence
1	32	162	20	19.8%
2	10	72	4	13.9%
3	16	77	9	20.8%
4	19	250	14	7.6%
5	31	651	26	4.8%
6	25	1049	16	2.4%

**Table 3:** Treatment and Survival.

Family	Common Name/Scientific Name	Tumor (s)	Survival in Months	Treatment ± Supportive Care
Boidae	Boa constrictor *Boa constrictor*	Benign carcinoid tumor	0.25	none
	Boa constrictor *Boa constrictor*	Soft tissue sarcoma	1	surgical excision only
	Rosy boa *Lichanura trivirgata*	Histiocytic sarcoma	7	surgical excision only
	Madagascar tree boa *Sanzinia madagascariensis*	Soft tissue sarcoma	22	surgical excision only
Colubridae	Western hog-nosed snake *Heterodon nasicus*	Hepatocellular carcinoma	10	none
	Western hog-nosed snake *Heterodon nasicus*	Soft tissue sarcoma	6	surgical excision *
	Eastern hog-nosed snake *Heterodon platirhinos*	Soft tissue sarcoma	15.5	none
	Kingsnake *Lampropeltis getula*	Soft tissue sarcoma, Hepatocellular adenocarcinoma	10.5	none
	Kingsnake *Lampropeltis getula*	Soft tissue sarcoma, Chromatophoroma (uncharacterized)	8	surgical excision only
	Common or northern watersnake *Nerodia sipedon*	Soft tissue sarcoma, Malignant chromatophoroma	5	surgical excision *
	Common or northern watersnake *Nerodia sipedon*	Malignant chromatophoroma	1	surgical excision only
	Common or northern watersnake *Nerodia sipedon*	Soft tissue sarcoma	0.5	surgical excision only
	Corn snake *Pantherophis guttatus*	Soft tissue sarcoma	2	none
	Corn snake *Pantherophis guttatus*	Soft tissue sarcoma	12	surgical excision and radiation and chemotherapy (Piroxicam) *
	Corn snake *Pantherophis guttatus*	Hemangiosarcoma	2	surgical excision only
	Corn snake *Pantherophis guttatus*	Soft tissue sarcoma	108	surgical excision only *
	Corn snake *Pantherophis guttatus*	Soft tissue sarcoma	18	surgical excision and radiation
	Corn snake *Pantherophis guttatus*	Soft tissue sarcoma	5.5	surgical excision and chemotherapy (Cyclophosphamide and Piroxicam)
	Ratsnake *Pantherophis obsoletus or alleghaniensis*	Colonic adenocarcinoma	7	surgical excision only *
	Ratsnake *Pantherophis obsoletus or alleghaniensis*	Chondrosarcoma	15	none
	Ratsnake *Pantherophis obsoletus or alleghaniensis*	Leukemia	1	none
	Ratsnake *Pantherophis obsoletus or alleghaniensis*	Osteosarcoma	6	supportive care only *
	Ratsnake *Pantherophis obsoletus or alleghaniensis*	Soft tissue sarcoma	4	surgical excision only
	Gophersnake(or) Bullsnake *Pituophis catenifer*	Hepatocellular carcinoma	1	none
	Black-necked gartersnake *Thamnophis cyrtopsis*	Malignant chromatophoroma	5.5	surgical excision only
	Plains gartersnake *Thamnophis radix*	Malignant chromatophoroma	13	surgical excision and radiation
	Plains gartersnake *Thamnophis radix*	Malignant granulosa cell tumor	1	surgical excision only
	Common gartersnake *Thamnophis sirtali*	Squamous cell carcinoma	9	not known
Elapidae	Ringhals cobra *Hemachatus haemachatus*	Lymphoma	0.75	none
	False water cobra *Hydrodynastes gigas*	Renal and Pancreatic adenocarcinoma	71	surgical excision only
	Red spitting cobra *Naja pallida*	Renal carcinoma/adenocarcinoma	35	surgical excision only
	King cobra *Ophiophagus hannah*	Lymphoma	11	chemotherapy only (Lomustine)
Pythonidae	Children’s python *Antaresia childreni*	Lymphoma, Soft tissue sarcoma	3	surgical excision only
	Carpet python *Morelia spilota*	Esophageal carcinoma	2.5	surgical excision only
	Green tree python *Morelia viridis*	Squamous cell carcinoma	7	not known
	Burmese python *Python bivittatus*	Soft tissue sarcoma	1	surgical excision only
	Ball python *Python regius*	Squamous cell carcinoma	84	surgical excision and chemotherapy (Carboplatin) *
Viperidae	Cottonmouth *Agkistrodon piscivorus*	Soft tissue sarcoma	0.25	surgical excision only
	Eyelash viper *Bothriechis schlegelii*	Soft tissue sarcoma	16	surgical excision only
	Eyelash viper *Bothriechis schlegelii*	Soft tissue sarcoma	5	none
	Eastern diamond-backed rattlesnake *Crotalus adamanteus*	Lymphoma	20	surgical excision and chemotherapy (Cyclophosphamide)
	Eastern diamond-backed rattlesnake *Crotalus adamanteus*	Squamous cell carcinoma	18.5	surgical excision only
	Eastern diamond-backed rattlesnake *Crotalus adamanteus*	Hemangiosarcoma, Sertoli cell tumor (uncharacterized)	7	surgical excision only
	Sidewinder *Crotalus cerastes*	Chromatophoroma (uncharacterized), Hepatobiliary adenoma	1	none
	Timber rattlesnake *Crotalus horridus*	Ovarian carcinoma	0.25	none
	Timber rattlesnake *Crotalus horridus*	Soft tissue sarcoma	0.25	none
	Timber rattlesnake *Crotalus horridus*	Soft tissue sarcoma	1	surgical excision only
	Timber rattlesnake *Crotalus horridus*	Lipoma	24	surgical excision only
	Rock rattlesnake *Crotalus lepidus*	Malignant chromatophoroma, Hepatobiliary adenoma	1	none
	Prairie rattlesnake *Crotalus viridis*	Hepatocellular carcinoma, Soft tissue sarcoma	1	none
	South american bushmaster *Lachesis muta*	Hepatocellular carcinoma/adenocarcinoma	3	none
	Eastern massasauga *Sistrurus catenatus*	Osteosarcoma	3.5	surgical excision *
	Sumatran pit viper *Trimeresurus sumatranus*	Lymphoma	0.36	none

* A nonsteroidal anti-inflammatory (NSAID) was also used less than 5 days for pain control.

**Table 4:** Predictor variable values of snakes that had a significant association with tumor versus non-tumor causes of death based on boosting and comparing each variable’s modeled outcome effect to null model distribution of effects generated from modeling done with permutated cause of death outcomes (*p* < 0.05).

Predictor Variable	Predictor Variable Value	Sample Size ^a^	Outcome Effect ^b^
Type of Neoplasm	Malignant	112	0.71
Type of Neoplasm	Benign	9	−0.71
Metastasis (Yes/No)	Yes	56	0.060
Metastasis (Yes/No)	Unknown Metastasis	2	0.060
Metastasis (Yes/No)	No	63	−0.055
Tumor Type	Chromatophoroma	4	−0.20

^a^ This is a subset of clinical case counts for which there were specific values reported across the set of eight predictor variables with a determined outcome of death due to tumor or death due to another cause. In the case of multiple tumor types and status of being malignant or benign, the tally and modeling is based on random sampling of a tumor type and its associated status for each clinical case. ^b^ Outcome effects are relative to death due to tumor or death due to another cause with each clinical case scored as +1 and 0 respectively.

## Data Availability

Deidentified data are available through an approved research request to the Exotic Species Cancer Research Alliance (https://escra.cvm.ncsu.edu/ (accessed on 18 January 2022)).
